# When to Measure Plasma Homocysteine and how to Place it in Context: The Homocystinurias

**DOI:** 10.34763/jmotherandchild.20202402si.2016.000007

**Published:** 2020-10-02

**Authors:** Martina Huemer

**Affiliations:** 1 Division of Metabolism, Children’s Research Center, University Children’s Hospital Zürich, Zürich, Switzerland; 2 Department of Paediatrics, Landeskrankenhaus Bregenz, Bregenz, Austria

**Keywords:** re-methylation, cystathionine beta synthase deficiency, MTHFR deficiency, vitamin B12, cobalamin, gastric intrinsic factor, Imerslund-Graesbeck syndrome, transcobalamin, TCN2

## Abstract

This review presents clinical patterns that should trigger homocysteine measurement in blood, as well as the further diagnostic work-up focused on inborn errors of metabolism and disorders of vitamin B12 (cobalamin) absorption and supply. The numerous conditions (e.g. cardiovascular disease, Alzheimer’s disease) for which mild-to-moderate hyperhomocysteinaemia caused by genetic polymorphisms or acquired reasons is considered a risk factor are beyond the scope of this review.

Homocysteine is a sulphur-containing amino acid, which is derived from the amino acid methionine. Homocysteine is either trans-sulphurated to form cystathionine and then cysteine, or re-methylated to methionine. The trans-sulphuration reaction depends on the enzyme cystathionine beta synthase and its cofactor vitamin B6. The re-methylation reaction not only involves the enzymes methionine synthase and methionine synthase reductase but also depends on the cofactor cobalamin and on the provision of methyl groups from the folate cycle. Because the homocysteine–methionine cycle provides for the vast majority of methyl groups in the body, it is central to numerous pathways that depend on methyl group supply, such as creatine synthesis or DNA methylation. Based on this premise, the severity of clinical presentations of inborn errors of metabolism, such as classical homocystinuria or the cobalamin C (cblC) defect, affecting this pathway is unsurprising.

## Introduction: Metabolic and Absorption Pathways

Homocysteine (Hcy) is a sulphur-containing amino acid that is derived from the essential amino acid methionine (Met). Met is metabolised by Met-adenosyltransferase (MAT I/III) to *S*-adenosylmethionine (AdoMet). Multiple methyltransferases are recipients of the methyl groups generated by the next step, the conversion of AdoMet to *S*-adenosylhomocysteine (AdoHcy). AdoHcy is transformed to Hcy, which is either irreversibly converted to cystathionine and thereafter cysteine by cystathionine beta synthase (CBS) and its cofactor vitamin B6 (the trans-sulphuration pathway) or re-methylated to Met by methionine synthase (MS; coded by the *MTR* gene), which depends on methionine synthase reductase (MSR; *MTRR*), the cofactor cobalamin (Cbl; synonym: vitamin B12) and methyl groups from the folate cycle delivered by 5-methyltetrahydrofolate (5-MTHF). Total Hcy (tHcy) in blood is elevated if the transsulphuration or the re-methylation pathway is disrupted (1, 2). If trans-sulphuration fails, Hcy is re-methylated to Met in greater amounts. Therefore, the biochemical fingerprint of CBS deficiency or classical homocystinuria is elevated Hcy in the presence of high Met and often low cystathionine (3, 4).

**Figure 1:**
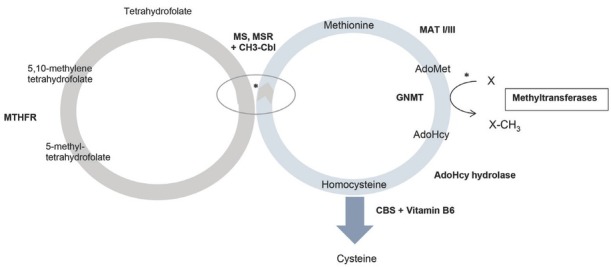
Simplified methionine-homocysteine pathway. AdoMet, S-adenosylmethionine; AdoHcy, S-adenosylhomocysteine; MTHFR, 5,10 methylenetetrahydrofolate reductase; MS, methionine synthase; CH3-Cbl, methylcobalamin; GNMT, glycine N-methyltransferase ; CBS, cystathionine β-synthase; MAT I/III, methionine adenosyltransferase. * methyl group transfer

Defective re-methylation of Hcy to Met results in both low AdoMet and elevated AdoHcy and thus causes impaired methyl (or ‘one-carbon’) group supply for numerous methylation reactions, e.g. creatine synthesis or DNA methylation, a regulatory mechanism involved in epigenetic processes (2) (Figure 1).

**Figure 2:**
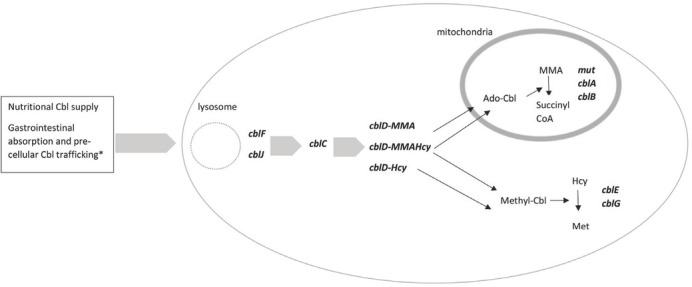
Simplified overview of the intracellular processing of Cbl and its intracellular metabolites adenosyl-Cbl and methyl-Cbl. In cblF and cblJ disease, liberation of Cbl from the lysosome and processing to the MMACHC gene product (cblC), which decyanides Cbl, fails. Depending on the locus of the mutation in the MMACHD gene, cblD disease may either affect the synthesis of both adenosyl-and methyl-Cbl or of only one of the pathways. Adenosyl-Cbl is the cofactor for intramitochondrial MMA metabolism by mutase and the cblA and cblB proteins. Methyl-Cbl is cofactor for the remethylation of Hcy to Met by MS (CblG) and MSR (cblE). *Main disorders in this pathway are gastric intrinsic factor deficiency (GIF), Imerslund Graesbeck syndrome (AMN; CUBN) and transcobalamin deficiency (TCN2).

The re-methylation disorders encompass inborn errors that directly affect the enzymes MS (*MTR*; in the cblG defect) or MSR (*MTRR*, in the cblE defect), as well as deficiency of methyltetrahydrofolate reductase (MTHFR; *MTHFR*), in

which the body is deprived of the methyl group-providing substrate 5-MTHF from the folate cycle. Elevated tHcy (and eventually low Met) is the biochemical fingerprint of the cblE and cblG defects, of MTHFR deficiency and of the cblD-Hcy (*MMADHC*) defect (5). As Cbl is an essential cofactor in the re-methylation reaction, inborn errors of absorption or intracellular processing of Cbl also cause defective re-methylation. Gastric intrinsic factor (*GIF)* deficiency, Imerslund-Graesbeck syndrome (*AMN; CUBN*) and transcobalamin deficiency (*TCN2)* are inborn disorders of Cbl absorption; and the cblF (*LMBRD1)*, cblJ *(ABCD4)*, cblC *(MMACHC)* and cblD-MMAHcy (*MMADHC*) defects impair the shared intracellular processing pathway of Cbl. These conditions affect not only re-methylation but also the intra-mitochondrial degradation of methylmalonic acid (MMA), to which Cbl is also essential. Elevated tHcy (and eventually low Met) in the presence of high MMA is the biochemical fingerprint of this group of disorders (Figure 2).

Severely impaired Cbl supply due to nutritional deprivation or acquired conditions hampering absorption (e.g. perniciousanaemia) also deprive the pathways of both adenosyl- and methyl-Cbl and can mimic the clinical and biochemical presentation of inborn errors of Cbl metabolism (Table 1) (6, 7, 8).

**Table 1:** Inborn errors and acquired conditions associated with elevated tHcy in blood

	*Gene*	*MIM code*	*MMA*	*Cbl*
^$^CBS deficiency (classical homocystinuria)	*CBS 21q22.3*	236200	n	^*^
***Acquired conditions resulting in decreased cobalamin supply / absorption***				
^#^Nutritional Cbl deficiency including neonatal due to maternal Cbl deficiency	-	-	↑	↓
^#^IF / gastric parietal cell antibodies	-	-	↑****	↓
^#^Gastric intrinsic factor deficiency	*GIF* 11q12.1	261000	↑	often ↓
***Inborn errors of cobalamin absorption***				
^#^Imerslund-Gräsbeck syndrome	*AMN* 14q32.32 *CUBN* 10p13	261100	↑	often ↓
^#^Transcobalamin deficiency	*TCN2* 22q12.2	275350	↑	^*^
***Combined inborn errors of remethylation***				
^#^cblF defect	*LMBRD1* 6q13	277380	↑	^*^
^#^cblJ defect	*ABCD4* 14q24.3	614857	↑	^*^
^#^cblC defect	*MMACHC* 1p34.1	277400	↑	^*^
^#^cblD-MMAHcy defect	*MMADHC* 2q23.2	277410	↑	^*^
***Isolated inborn errors of remethylation***				
^#^cblD-Hcy defect	*MMADHC* 2q23.2	277410	n	^*^
^#^cblE defect	*MTRR* 5p15.31	236270	n	^*^
^#^cblG defect	*MTR* 1q43	250940	n	^*^
^#^MTHFR deficiency	*MTHFR* 1p36.22	236250	n	^*^

$ high methionine;

* Cbl is expected to be normal (but may be affected by acquired conditions such as decreased su pply). In haptocorrin deficiency, transcobalamin receptor deficiency, MTHFD1 deficiency, and the X-chromosomally inherited HCFC1 defect, tHcy may be but is not consistently elevated. In hypermethioninemias caused by MAT I/III, GNMT, SAHH or ADK deficiencies, tHcy is usually normal or only mildly elevated (usually below 50 μmol/L ) [6, 19, 20].

# low or normal methionine;

### Clinical Situations That Should Prompt Measurement of tHcy: The Natural History

The clinical presentation of the homocystinurias is widespread in two dimensions. One is age – although the majority of patients come to clinical attention as neonates or infants, homocystinurias may present any time throughout life. The second dimension is the clinical presentation – homocystinurias are clinically heterogeneous, multi-system disorders.

Homocystinurias should be considered as a differential diagnosis in a neonate with acidosis, failure to thrive, neutropenia, seizures and irritability; in a child with cognitive impairment, lens dislocation and a marfanoid habitus; in an adolescent with atypical haemolytic-uraemic syndrome and acute renal failure; or in an adult with psychiatric symptoms, dementia, subacute combined degeneration of the spinal cord and signs of peripheral neuropathy or thromboembolism (7, 9, 10, 11).

Acquired re-methylation impairment due to insufficient Cbl supply should be suspected in any infant developing symptoms of neurocognitive decline, eventually microcephaly, feeding difficulties, failure to thrive and irritability, typically in the second-to-third quarter of the first year. As the only natural Cbl sources for humans are animal products, the offspring of breastfeeding mothers adhering to a vegan lifestyle are especially prone to nutritional Cbl deficiency. Long-standing Cbl deficiency in an infant is a serious condition that may cause irreversible neurological damage (7, 12, 13).

The homocystinurias share the hallmark of damage to the central nervous system. Feeding difficulties, failure to thrive, irritability, seizures, cognitive impairment, movement disorders, neuropathy, white matter disease and hypomyelination, as well as combined degeneration of the spinal cord, should alert the clinician to re-methylation disorders. Early-onset (before Month 12 of life) Cbl-related inborn errors of re-methylation (especially the most frequent cblC defect) often also cause retinopathy (retinitis pigmentosa) and optic atrophy, resulting in a decline of visual acuity and even blindness. This specific eye damage does not occur in patients with MTHFR deficiency or in late-onset patients with Cbl-related inborn errors of re-methylation. In all re-methylation defects, children may present with failure to thrive and developmental delay, while in adolescent or adult patients, psychiatric problems, neuropathy and dementia are leading symptoms (5, 14). Classical homocystinuria patients show intellectual, psychiatric and behavioural problems, as well as movement disorders and sometimes seizures. Their leading eye problem is ectopia lentis, which is predominantly seen in untreated young, severely affected patients (3, 4).

Homocystinurias are in most cases multi-system disorders with a broad range of additional, non-neurological symptoms. A marfanoid skeletal phenotype with excessive height and limb length, arachnodactyly and thromboembolism are characteristic manifestations of CBS deficiency. Clinical liver disease is not a hallmark of CBS deficiency, but fatty liver or liver fibrosis has been noted (3, 4).

In re-methylation disorders, thromboembolism occurs – like in CBS deficiency- mostly in adolescence or adulthood. In contrast to CBS deficiency however, re-methylation disorders are also complicated by micro-angiopathy, which may at any age manifest either as chronic glomerular or tubulo-interstitial disease, acute renal failure under the clinical picture of atypical haemolytic-uraemic syndrome, pulmonary arterial hypertension or hydrocephalus (the latter mainly in newborns). Megaloblastic anaemia, neutropenia or pancytopenia is frequent; cardiac malformations and cardiomyopathy occur sometimes, while liver disease is quite rare in re-methylation defects (5, 9, 10, 14). Table 2 displays an overview of the most frequent symptoms of the homocystinurias at different ages (3, 4, 5, 9, 10, 14, 15, 16).

**Table 2:** Symptoms at disease presentation by age in remethylation disorders (a) and CBS deficiency (b) in estimated order of frequency. There is wide range of ages at presentation and spectrum of severity, from asymptomatic individuals to severely affected patients with multi-system disease.

(a) Remethylation disorders
Early onset (<12 months)
Feeding difficulties / failure to thrive
Muscular hypotonia
Developmental / cognitive impairment
Seizures
Eye disease (nystagmus, visual impairment)^*^ Hydrocephalus
Acute metabolic decompensation
Cardiac disease (cardiac malformation; cardiomyopathy
Atypical haemolytic uraemic syndrome
Behavioural problems
Movement disorders
Stroke / thromboembolic event
Anaemia/thrombocytopenia or pancytopenia, megaloblastosis
Chronic renal failure
Pulmonary hypertension
Late onset (>12 months)
Failure to thrive / weight loss / feeding problems
Developmental / cognitive impairment
Seizures
Muscular hypotonia / muscle weakness
Thromboembolism / stroke / pulmonary embolism
Psychiatric disease
Movement disorder
Myelopathy
Atypical haemolytic uraemic syndrome
Acute metabolic decompensation
Chronic renal failure
Cardiac disease
[5, 9, 10, 14]
(b) CBS deficiency (classical homocystinuria)^#^
Ectopia lentis and/or severe myopia
Developmental delay/intellectual disability
Thromboembolic events
Excessive height and length of the limbs (‘marfanoid’ habitus)
Osteoporosis and bone deformities (pectus excavatum or carinatum, genu
valgum, scoliosis)
Seizures, psychiatric and behavioural problems and extrapyramidal signs
[3,4, 15,32]

* The typical eye disease observed in Cbl-related remethylation disorders is generally limited to early onset forms and not present in MTHFR deficiency.

# Clinical manifestations are generally more severe in pyridoxine non-responsive disease.

### Work-up of the Patient with Hyperhomocysteinaemia

Generally, tHcy concentrations increase with age; they are lowest in young children and in adults, in pre-menopausal women. If elevated tHcy levels are reported by the laboratory, primarily, common disorders and problems causing secondary, often milder elevation of tHcy such as insufficient supply of Vitamin B12, folate – or (rarely) vitamin B6 – renal insufficiency or hypothyroidism need to be excluded. In CBS deficiency, and the re-methylation disorders, tHcy mostly exceeds 70–100 mmol/L (3, 5).

**Figure 3:**
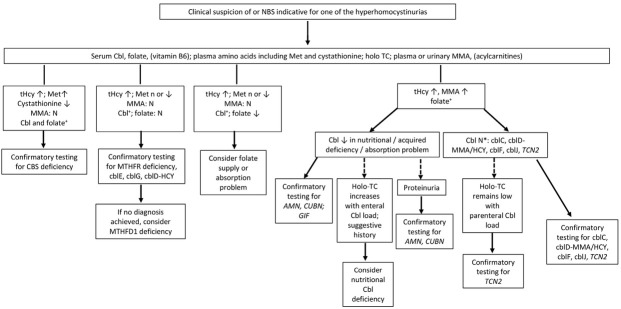
Diagnostic algorithm for patients with elevated tHcy. Dotted arrows indicate investigations that may be helpful but are not confirmatory. +Parameters do not correspond with the biochemical phenotype and are expected to be normal but may be low for unrelated reasons. *low Cbl has been observed in some patients with combined remethylation disorders for unclear reasons

For further work-up of high levels of tHcy, measurement of the following biochemical parameters is helpful and facilitates focused confirmatory studies: folate, Cbl (eventually vitamin B6), holo-transcobalamin (holo-TC), MMA in blood or urine (urinary MMA is sufficient if renal function is normal) and plasma amino acids (including Met and cystathionine). Propionylcarnitine (C3) is elevated and some acylcarnitine ratios are perturbed in the presence of MMA; thus, the acylcarnitine pattern may be used (and is often used by newborn screening [NBS] programmes) as a preliminary screening parameter if MMA measurement would not immediately be available. Following careful evaluation of biochemical findings, nowadays, molecular genetic studies are the method of choice to confirm an inborn error of metabolism associated with high tHcy. Enzymatic studies, however, still have their place if genetic studies are unavailable or yield inconclusive results (17) (Figure 3).

Some other very rare inborn diseases that may also affect tHcy must be kept in mind as differential diagnoses. THcy may be, but is not consistently, elevated in haptocorrin deficiency, transcobalamin receptor deficiency (6, 7, 8), deficiency of a trifunctional enzyme (5,10-methylenetetrahydrofolate dehydrogenase, 5,10-methenyltetrahydrofolate cyclohydrolase, 10-formyltetrahydrofolate synthetase) in MTHFD1 deficiency (18), as well as in defects of the transcriptional co-regulator HCFC1 (19) or THAP11, a thanatos-associated protein (THAP) domain-containing transcription factor (20). In the hypermethioninaemias caused by deficiencies of MAT I/III, glycine *N*-methyltransferase (GNMT), AdoHcy hydrolase (SAHH) or adenosine kinase (ADK), tHcy is usually normal or only mildly elevated (usually <50 mmol/L) (21, 22).

Isolated defects of MMA metabolism related to mutase dysfunction, as well as the cblD-MMA, cblA and cblB defects, display normal tHcy concentrations (6).

### Treatments and Contraindications

Treatment, especially if initiated early, is highly successful in CBS deficiency. Some mutations allow for biochemical (reduction of tHcy) and clinical responsiveness to treatment with the cofactor vitamin B6 (‘B6-responsive’ CBS). B6-responsive patients generally have a milder disease course. At diagnosis of CBS deficiency, a standardised B6 test, as suggested by Morris et al. (4), should be undertaken in every patient in whom a B6-unresponsive form is not completely evident, e.g. by family studies.

B6-unresponsive patients, as patients with only partial responsiveness, rely on dietary treatment with restriction of natural protein intake to minimise Met load to consecutively reduce tHcy. Betaine is available as a registered drug for the homocystinurias. It lowers tHcy by opening an alternative remethylation pathway of Hcy to Met via the enzyme betaine homocysteine methyl transferase. Met must be carefully monitored, because with very high Met levels, single cases of brain oedema have been reported, which responded favourably to betaine withdrawal and consecutive lowering of Met. Thus, it is recommended to avoid plasma Met concentrations exceeding 800 (21) or 1000 mmol/L (4). Currently, enzyme replacement therapy for CBS is under development, and it will be interesting to follow up on both the options that this new treatment may open and its limitations (23).

For patients with MTHFR deficiency, betaine is the drug of choice; early treatment with betaine even prevents clinical symptoms (24), and thus NBS should definitely be considered in populations with relevant incidence of the disease (25, 26). In MTHFR deficiency, shortage of 5-MTHF in the brain has been observed, and although this deficiency is of unclear significance, folic acid should not be given since it may aggravate the shortage of 5-MTHF in the brain due to competitive transport at the blood–brain barrier. Significant clinical effects of the often-used supplementation with folinic acid are yet to be proven (27).

In the Cbl-related re-methylation disorders, parenteral hydroxo-Cbl (OH-Cbl) is the mainstay of treatment. In the cblC defect, the most frequent of these disorders with several hundreds of patients, it has been clinically observed that cyano-Cbl, an easily available orally applicable Cbl preparation, is ineffective (28). This observation has been explained several years later when it could be shown that the *MMACHC* gene product decyanates Cbl (29). Since other Cbl-dependent re-methylation disorders are clinically undistinguishable from the cblC disease, OH-Cbl has consequentially also been the drug of choice in these disorders, and experience with other Cbl preparations is missing. Carnitine, given to remove MMA from the system in combined disorders with both homocystinuria and MMA elevation, and folic or folinic acid, used to prevent deficiencies and optimise the folate cycle, are often applied but have no proven positive effect (5, 17).

In all disorders of re-methylation, nitrous oxide must not be given as it arrests the re-methylation pathway and leads to lethal complications in patients (17, 30, 31). In contrast to CBS deficiency, patients with re-methylation disorders should not be treated with protein-restrictive diet as low protein intake aggravates shortage of both Met and methyl groups and may aggravate the clinical status (5, 17, 32).

Disorders of gastrointestinal B12 absorption and transportation (Imerslund-Graesbeck disease, GIF deficiency or TCN2 deficiency) are treated with parenteral vitamin B12 preparations, and if detected and treated early, they have a good prognosis. Nutritional deficiency, either in an individual directly or mediated in a newborn by Cbl deficiency in its mother, must be treated immediately, and the effect of treatment is to be monitored clinically and biochemically (normalisation of tHcy and MMA being the targets). Generally, for treatment start, parenteral application is recommended as is counselling on balanced nutrition. After refilling the stores with parenteral treatment, a switch to oral treatment and/or food containing sufficient amounts of Cbl may be considered, patient’s adherence provided (6, 7, 8).

### Outcome

Studies from Qatar and Ireland, countries with large communities of B6-unresponsive CBS patients have clearly demonstrated the efficacy of early treatment and provide convincing evidence for the effectiveness of NBS for CBS deficiency. Early treatment prevents severe symptoms, such as lens dislocation, thromboembolism and neurocognitive impairment (3, 16).

There is less data available on MTHFR deficiency, but this disease also seems to be well treatable. The earlier the initiation of betaine, the better is the outcome, and neurocognitive symptoms may even be prevented (24). For the Cbl-related re-methylation defects, most evidence is available from cblC patients. It is well established that late-onset patients (>12 months), mainly adolescents and adults who usually unfortunately have the longest time to diagnosis due to the heterogeneous clinical presentation, profit greatly from treatment. However, lost function due to belated treatment may not be regained. In early-onset re-methylation disorders, survival, renal disease, thromboembolic events and other organ manifestations are greatly improved by treatment. Unfortunately however, even in children treated antenatally or from birth, neurocognitive and behavioural problems, as well as the typical eye disease with retinopathy and optic nerve atrophy, do not respond to standard treatment (5, 14, 33). Currently, there is accumulating evidence that high-dose OH-Cbl may be associated with better outcomes (34, 35), but systematic studies are necessary to prove this effect.

GIF deficiency, Imerslund-Graesbeck syndrome and TCN2 deficiency have a good prognosis when treated early and appropriately (8). Nutritional deficiencies, especially in the neonate, may result in severe neurological disease and failure to thrive. If treated early, symptoms resolve, but long-standing deficiency due to delays in diagnosing the condition often leaves the patient with neurocognitive sequelae (12, 13).

## Conclusions

The homocystinurias are treatable disorders, which clinically generally involve the central nervous and multiple other organ systems. The homocystinurias often become clinically evident early in life but may also do so any time later in life. Early treatment may prevent or at least alleviate the burden of the disease. Clinical consideration of these disorders, tHcy measurement and consecutive work-up and allocation of the patient to a specific defect makes a significant difference for affected patients and their families.
